# Integrated Transcriptomic and Metabolomic Analysis of Exogenous NAA Effects on Maize Seedling Root Systems under Potassium Deficiency

**DOI:** 10.3390/ijms25063366

**Published:** 2024-03-16

**Authors:** Dongying Zhou, Yuanchun Zhang, Qiqi Dong, Kai Wang, He Zhang, Qi Du, Jing Wang, Xiaoguang Wang, Haiqiu Yu, Xinhua Zhao

**Affiliations:** 1 College of Agronomy, Shenyang Agricultural University, Shenyang 110866, China; zhoudy@stu.syau.edu.cn (D.Z.); 2021240384@stu.syau.edu.cn (Y.Z.); qiqidong77_syau@163.com (Q.D.); wangkai05@caas.cn (K.W.); zhanghe@syau.edu.cn (H.Z.); 2022200064@stu.syau.edu.cn (Q.D.); jingwang602@syau.edu.cn (J.W.); wxg@syau.edu.cn (X.W.); yuhaiqiu@syau.edu.cn (H.Y.); 2 National Nanfan Research Institute (Sanya), Chinese Academy of Agricultural Sciences, Sanya 572000, China; 3 Coastal Agriculture Institute, Hebei Academy of Agricultural and Forestry Sciences, Tangshan 063299, China

**Keywords:** auxin, potassium deficiency, root development, potassium uptake, lignin synthesis

## Abstract

Auxin plays a crucial role in regulating root growth and development, and its distribution pattern under environmental stimuli significantly influences root plasticity. Under K deficiency, the interaction between K^+^ transporters and auxin can modulate root development. This study compared the differences in root morphology and physiological mechanisms of the low-K-tolerant maize inbred line 90-21-3 and K-sensitive maize inbred line D937 under K-deficiency (K^+^ = 0.2 mM) with exogenous NAA (1-naphthaleneacetic acid, NAA = 0.01 mM) treatment. Root systems of 90-21-3 exhibited higher K^+^ absorption efficiency. Conversely, D937 seedling roots demonstrated greater plasticity and higher K^+^ content. In-depth analysis through transcriptomics and metabolomics revealed that 90-21-3 and D937 seedling roots showed differential responses to exogenous NAA under K-deficiency. In 90-21-3, upregulation of the expression of K^+^ absorption and transport-related proteins (proton-exporting ATPase and potassium transporter) and the enrichment of antioxidant-related functional genes were observed. In D937, exogenous NAA promoted the responses of genes related to intercellular ethylene and cation transport to K-deficiency. Differential metabolite enrichment analysis primarily revealed significant enrichment in flavonoid biosynthesis, tryptophan metabolism, and hormone signaling pathways. Integrated transcriptomic and metabolomic analyses revealed that phenylpropanoid biosynthesis is a crucial pathway, with core genes (related to peroxidase enzyme) and core metabolites upregulated in 90-21-3. The findings suggest that under K-deficiency, exogenous NAA induces substantial changes in maize roots, with the phenylpropanoid biosynthesis pathway playing a crucial role in the maize root’s response to exogenous NAA regulation under K-deficiency.

## 1. Introduction

Potassium (K), as one of the most prevalent inorganic cations in plants, plays a pivotal role in essential physiological processes such as photosynthesis, enzyme activation, substance synthesis, and transport [1,2]. Despite the high cytoplasmic concentrations of K^+^ (around 100 mM), natural soil typically contains low K^+^ concentrations (0.1~1 mM) [3,4,5]. In modern agriculture, the extensive use of fertilizers has mitigated mineral nutrient deficiencies in crop growth [6,7,8]. However, challenges such as environmental pollution, ecological degradation, and raw material scarcity pose significant threats to sustainable agricultural development. Therefore, identifying low-K-tolerant crops and researching potential low-K tolerance mechanisms are crucial for advancing crop improvement.

Roots are a crucial organ for nutrient absorption; both their morphology and physiological changes significantly impact the absorption and utilization of nutrients [9]. When K is absorbed through the roots from the external environment, it is either stored in vacuoles or transported through the xylem to the aboveground parts. Subsequently, K-ions are translocated and distributed to different organs and tissues of plants by cells in the shoot via the vascular system [10,11,12]. However, variations in the response to K-deficiency among different varieties of crops are observed, often characterized by more flexible root structures, increased length and density of lateral roots, and root hairs in K-tolerant varieties, thereby widening the scope of nutrient absorption by the roots [13]. Additionally, in a study on soybean roots under K-deficiency, significant alterations were observed in the biosynthetic pathways of flavonoids and flavonols in K-tolerant varieties, accompanied by the upregulation of genes related to K-ion absorption and transport [14]. Similarly, transcriptomic studies on wheat revealed that K-tolerant varieties can selectively regulate genes related to the ethylene response, auxin response, and nutrient reserve in response to K deficiency [15].

Auxin, a vital hormone regulating plant growth and development, plays a crucial role in the distribution of roots in plants [16]. This distribution influences root apical meristem, post-injury regeneration, tropical root growth, and lateral root density [17,18]. Auxin plays a pivotal role in the plant response to K deficiency. Previous studies have indicated that both auxin-induced growth and proton secretion are heavily dependent on the extracellular K-ion concentration and K-ion uptake mediated by K-ion channels on the plasma membrane [16,19]. Among the four high-affinity K^+^ transport protein families, the KUP/HAK/KT family stands out as the most prominent in plants, with its members widely involved in K-ion transmembrane transport in fungi, bacteria, and plants [20]. Among them, the *HAK5* gene exhibits specific expression in roots and higher expression levels under K deficiency [2]. Interestingly, studies in *Arabidopsis* have revealed that Auxin Response Factor 2 (ARF2)acts as a transcriptional repressor, binding to the *HAK5* gene promoter under normal K^+^ levels to suppress *HAK5* expression. However, under K-deficiency conditions, ARF2 is phosphorylated, thereby alleviating its inhibitory effect on *HAK5*. *arf2*-mutant plants under K-deficiency and plants overexpressing *HAK5* exhibit similar phenotypes of K-tolerance in roots. However, the K-tolerance phenotype disappears in *arf2*-mutant plants upon knockout of the *HAK5* gene [21,22]. Furthermore, in sweet potatoes, genes related to auxin (*IbPIN1*, *IbPIN3*, *IbAUX1*, *IbIAA4*, and *IbIAA8*) were found to participate in regulating sweet potato lateral root formation and root architecture changes under K-deficiency treatment, promoting K uptake and biomass formation [23]. In our preliminary research, we found that, under K-deficiency, compared to those in the K-sensitive inbred line D937, the root length, root volume, and root surface area of the K-tolerant maize inbred line 90-21-3 significantly increased, accompanied by a significant increase in indole-3-acetic acid (IAA) content [24].

Given the crucial role of IAA in plant root nutrient uptake, previous research has demonstrated the alleviation of nutrient stress through the exogenous addition of IAA [25,26,27,28]. For example, exogenous IAA has been shown to regulate root architecture under phosphorus limitation, inhibiting primary root elongation and promoting the formation and proliferation of lateral and adventitious roots [29]. In our study, we aimed to comprehensively investigate the effects of exogenous NAA (1-naphthaleneacetic acid) on root growth and development, K^+^ uptake and utilization, physiological metabolism, and genes expression in maize seedlings under K-deficiency. The study’s objectives included (i) comparing the regulatory effects of exogenous NAA on the morphology and potassium uptake/utilization of different tolerant inbred maize lines; (ii) investigating differential changes in gene expression and metabolite accumulation in different tolerant varieties under K-deficiency with exogenous NAA treatment; and (iii) revealing key metabolic pathways, genes, and metabolites in exogenous NAA that regulate the adaptation of maize root systems to K-deficiency.

## 2. Results

### 2.1. Root Growth and Development

**Figure 1:**
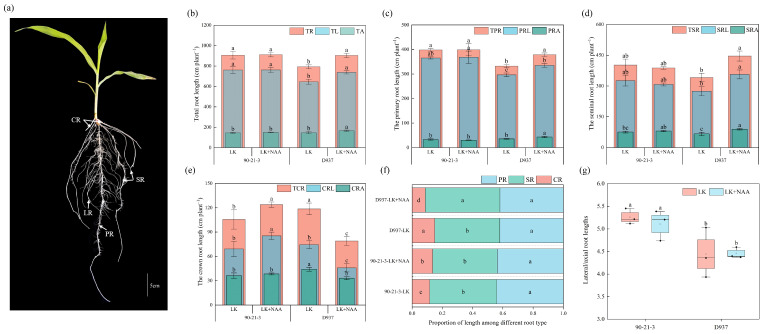
Effects of exogenous NAA on root development of 90-21-3 and D937 under low-K^+^ stress. (**a**) Root system architecture in maize seedling. (**b**) Total root length. (**c**) Primary root length. (**d**) Seminal root length. (**e**) Crown root length. (**f**) Proportion of length among different root types. (**g**) Lateral/axial root length. TR, total root length; TL, total lateral root length; TA, total axial root length; TPR, total primary root length; PRL, lateral root length of primary root; PRA, axial root length of primary root; TSR, total seminal root length; SRL, lateral root length of seminal root; SRA, axial root length of seminal root; TCR, total crown root length; CRL, lateral root length of crown root; CRA, axial root length of crown root; CR, crown root; LR, lateral root; PR, primary root; SR, seminal root. LK represents low potassium treatment, while LK + NAA represents low potassium stress with exogenous NAA application. Bars indicate SD. Different lowercase letters in the graphs indicate significant differences (*p* < 0.05).

Roots subjected to different treatments were analyzed according to root classification, as shown in Figure 1a. Our results showed that the addition of exogenous NAA (LK + NAA) significantly increased the total root length (TR) of the K^+^-sensitive inbred line D937 under K deficiency (LK) treatment. Specifically, the total lateral root length (TL) and total axial root length (TA) increased by 14.29% (from 645.81 to 738.10 cm) and 13.15% (from 146.50 to 165.76 cm), respectively (Figure 1b). Analysis of the primary root (TPR) and seminal root (TSR) lengths showed that the lateral root length (PRL) and axial root length (PRA) of the D937 primary root increased by 13.01% (from 296.64 to 335.50 cm) and 21.06% (from 35.73 to 43.26 cm), respectively, under LK + NAA treatment compared to LK (Figure 1c,d). Subsequently, the lateral root length (SRL) and axial root length (SRA) of the seminal root increased by 29.85% (from 274.67 to 356.67 cm) and 34.17% (from 66.63 to 89.40 cm), respectively. However, in 90-21-3, TR, TPR, and TSR showed less variation. Further determination of the crown root length (TCR) showed that the crown roots of 90-21-3 and D937 responded differently to LK + NAA treatment. In particular, 90-21-3 showed a significant increase in TCR, especially in the lateral root length (CRL) of the crown root, which increased by 23.34% (from 69.25 to 85.41 cm) (Figure 1e). On the contrary, the TCR of D937 was significantly reduced. The CRL and axial root length (CRA) showed significant reductions of 38.36% (from 74.50 to 45.92 cm) and 25.00% (from 44.13 to 33.10 cm) respectively. Analysis of the percentage of length of different types of root systems in maize seedlings showed that seminal and primary roots showed greater dominance (Figure 1f). The contribution of SR to the total root length of D937 was increased under LK + NAA treatment, and the contribution of CR to the total root length was increased in 90-21-3. Taking the ratio of total lateral root length to total axial root length, we found that LK + NAA treatment had no significant effect on the ratio of total lateral root length to total axial root length in both varieties. However, 90-21-3 consistently maintained a higher percentage of lateral root length than D937 (Figure 1g).

### 2.2. Root Growth and K^+^ Uptake

**Figure 2:**
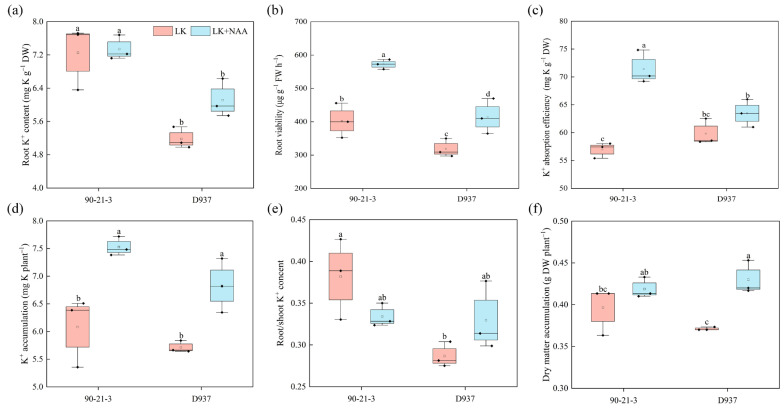
Effects of exogenous NAA on root growth and K^+^ uptake by 90-21-3 and D937 seedlings under K-deficiency. (**a**) Root K^+^ content. (**b**) Root viability. (**c**) K^+^ absorption efficiency. (**d**) K^+^ accumulation. (**e**) Root/shoot K^+^ content. (**f**) Dry matter accumulation. LK represents low potassium treatment, while LK + NAA represents low potassium stress with exogenous NAA application. Bars indicate SD. Different lowercase letters in the graphs indicate significant differences (*p* < 0.05).

Under K deficiency, the LK + NAA treatment had a positive effect on root elongation in D937, which could lead to an increase in K^+^ content in the roots (Figure 2a). Additionally, both the 90-21-3 and D937 varieties showed a significant increase in root viability under LK + NAA stimulation, with respective increments of 42.11% (from 402.71 to 572.27 μg g^−1^ h^−1^) and 30.13% (from 318.79 to 414.83 μg g^−1^ h^−1^) compared to the LK treatment (Figure 2b). Moreover, the roots’ K^+^ absorption efficiency significantly increased in both inbred lines, especially in 90-2-13, which experienced a 28.93% (from 58.04 to 74.83 mg g^−1^) increase (Figure 2c). The root K^+^ accumulation determination also revealed that the K^+^ accumulation of 90-21-3 and D937 seedlings significantly increased by 17.21% (from 6.38 to 7.48 mg) and 12.47% (from 5.64 to 6.35 mg), respectively, under the LK + NAA treatment (Figure 2d). The inclusion of NAA in the LK treatment led to a rise in K^+^ migration towards the roots in D937, but this increase was not statistically significant. Nevertheless, it significantly promoted dry matter accumulation (Figure 2e,f). However, this effect was not observed in 90-21-3.

### 2.3. The Transcriptomics of 90-21-3 and D937 Response to Exogenous NAA under K-Deficiency

**Figure 3:**
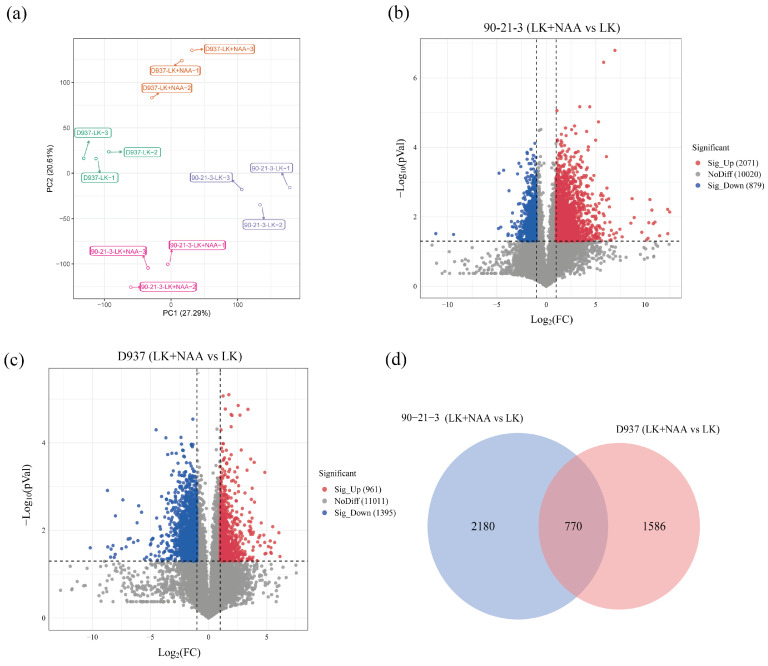
Transcriptome data of 90-21-3 and D937 roots in response to exogenous NAA under low K stress. (**a**) Principal component analysis (PCA) clustering based on the plants’ transcriptome data. (**b**,**c**) The volcano plot showed the differentially expressed genes (DEGs) of 90-21-3 and D937 under LK + NAA treatment compared with LK treatment. (**d**) Venn diagram showing DEGs for the groups 90-21-3 (LK + NAA vs. LK) and D937 (LK + NAA vs. LK). LK represents low potassium treatment, while LK + NAA represents low potassium stress with exogenous NAA application.

To comprehensively explore the molecular mechanisms governing exogenous NAA responses in 90-21-3 and D937 under K-deficiency conditions, we employed transcriptome sequencing to analyze the differentially expressed genes(DEGs) profiles in both 90-21-3 and D937 exposed to LK and LK + NAA treatments. Employing principal component analysis (PCA), we initially discerned significant transcriptomic variations among the 90-21-3-LK, 90-21-3-LK + NAA, D937-LK, and D937-LK + NAA groups (Figure 3a). Notably, the distinct segregation of maize inbred lines in response to LK and LK + NAA treatments highlighted the high repeatability and consistency within each treatment group. Our analysis identified a total of 12,970 and 13,367 DEGs in 90-21-3 and D937 under LK + NAA compared to LK conditions, comprising 2071 and 879 upregulated DEGs (|log2FC| > 1, *p*-value < 0.05), respectively, along with 961 and 1395 downregulated DEGs, respectively (Figure 3b,c). Remarkably, the number of DEGs in 90-21-3 exceeded that in D937, suggesting a broader response of the low-K-tolerant genotype to exogenous NAA in scenarios of K deficiency. Moreover, within 90-21-3, the prevalence of upregulated genes significantly outnumbered the downregulated ones compared to D937, revealing distinct patterns in their respective adaptive mechanisms.

### 2.4. Enrichment Analysis of Differentially Expressed Genes

**Figure 4:**
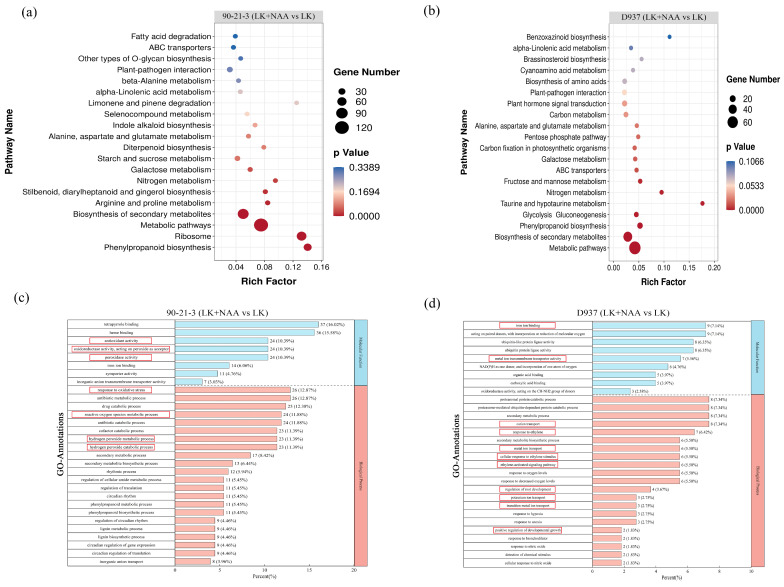
Enrichment analysis of the differentially expressed genes. (**a**,**b**) The top 20 Kyoto Encyclopedia of Genes and Genomes (KEGG) pathways were enriched in the DEGs of 90-21-3 (LK + NAA vs. LK) and D937 (LK + NAA vs. LK), respectively. (**c**,**d**) The top 30 Gene Ontology (GO) functional annotations were identified for the DEGs of 90-21-3 (LK + NAA vs. LK) and D937 (LK + NAA vs. LK), respectively. The red boxes indicate key GO functional items. LK represents low potassium treatment, while LK + NAA represents low potassium stress with exogenous NAA application.

To elucidate the pivotal biological pathways influenced by exogenous NAA in regulating the root system of maize seedlings, we conducted a Kyoto Encyclopedia of Genes and Genomes (KEGG) enrichment analysis of DEGs (|log2FC| ≥ 1.5) in both 90-21-3 and D937 genotypes. The KEGG enrichment analysis revealed significant enrichment of 8 out of the top 20 KEGG pathways in 90-21-3 (Figure 4a) and 14 pathways in D937 (Figure 4b). Notably, five pathways—phenylpropanoid biosynthesis, metabolic pathways, biosynthesis of secondary metabolites, nitrogen metabolism, and galactose metabolism—exhibited significant enrichment in both 90-21-3 (LK + NAA vs. LK) and D937 (LK + NAA vs. LK). However, distinct enrichments were observed in specific pathways: starch and sucrose metabolism, as well as Indole alkaloid biosynthesis pathways, were notably enriched in 90-21-3 (LK + NAA vs. LK), while the plant hormone signal transduction pathway exhibited significant enrichment in D937 (LK + NAA vs. LK). These findings from the KEGG enrichment analysis suggest that under K-deficiency and LK + NAA treatment, the root systems of 90-21-3 and D937 primarily adapt by modulating DEGs associated with metabolic pathways.

DEGs were categorized based on Biological Process, Molecular Function, and Cellular Components via Gene Ontology (GO) mapping (Figure 4c,d). The top 30 biological function items were ranked according to their *p*-values. Notably, significant enrichment of DEGs in Molecular Function and Cellular Components was observed in both 90-21-3 (LK + NAA vs. LK) and D937 (LK + NAA vs. LK). Specifically, within the Molecular Function category, a higher abundance of DEGs in 90-21-3 (LK + NAA vs. LK) exhibited enrichment in heme binding (GO:0046906) and tetrapyrrole binding (GO:0020037), while D937 (LK + NAA vs. LK) showcased enrichment in iron ion binding (GO:0005506) and acting on paired donors, with the incorporation or reduction of molecular oxygen (GO:0016705). In terms of the Cellular Component, 90-21-3 (LK + NAA vs. LK) presented more concentrated DEGs in the antibiotic metabolic process (GO:0016999) and response to oxidative stress (GO:0006979). In contrast, D937 (LK + NAA vs. LK) displayed enriched DEGs in the proteasomal protein catabolic process (GO:0010498), proteasome-mediated ubiquitin-dependent protein catabolic process (GO:0043161), secondary metabolic process (GO:0019748), and cation transport (GO:0006812). Further analysis revealed that DEGs in 90-21-3 (LK + NAA vs. LK) primarily enriched antioxidant-related functional items such as the hydrogen peroxide catabolic process (GO:0042744), hydrogen peroxide metabolic process (GO:0042743), reactive oxygen species metabolic process (GO:0072593), response to oxidative stress (GO:0006979), peroxidase activity (GO:0004601), oxidoreductase activity acting on peroxide as acceptor (GO:0016684), and antioxidant activity (GO:0016209). Conversely, D937 showcased more enrichment in metal ion binding and transport, including functions related to transition metal ion transport (GO:0000041), potassium ion transport (GO:0006813), metal ion transport (GO:0030001), cation transport (GO:0006812), metal ion transmembrane transporter activity (GO:0046873), and iron ion binding (GO:0005506) (Supplemental Table S2). Additionally, D937 displayed enrichment in ethylene-related functions such as ethylene-activated signaling pathway (GO:0009873), cellular response to ethylene stimulus (GO:0071369), and response to ethylene (GO:0009723), along with functions related to root development including positive regulation of developmental growth (GO:0048639) and regulation of root development (GO:2000280) (Supplemental Table S2). GO enrichment analysis highlighted substantial differences in the root regulatory mechanisms between 90-21-3 and D937 under LK + NAA treatment.

Our investigation focused on the DEGs associated with K-ion uptake and transport in the root system of K-deficiency maize seedlings subjected to exogenous NAA treatment. We identified 16 significantly altered genes (Supplemental Table S3). In 90-21-3, a majority of genes exhibited heightened expression levels. Specifically, pronounced upregulation was observed in genes related to proton-exporting ATPase activity (Zm00001d002006, Zm00001d026490, Zm00001d015426, Zm00001d028436, and Zm00001d000233). Additionally, seven genes associated with K^+^ transport (Zm00001d003859, Zm00001d003861, Zm00001d008424, Zm00001d018799, and Zm00001d040157), as well as those involved in K^+^ channel activity (Zm00001d011473 and Zm00001d038618), all of which displayed elevated expression levels. In contrast, within D937, the majority of DEGs exhibited significant downregulation, except for one gene (Zm00001d003213) associated with K^+^ channel activity, demonstrating an upregulated expression pattern. This divergent response to exogenous NAA treatment under low-K conditions between the 90-21-3 and D937 lines emphasizes substantial differences in their respective reactions. This disparity underscores the varying impact of exogenous NAA on gene expression related to K^+^ dynamics within K-deficiency maize seedlings of differing genotypes.

### 2.5. Quantitative Real-Time PCR Validation

We conducted quantitative real time PCR (qRT-PCR) analysis on 16 established transcripts to validate the accuracy of the sequencing data (Supplementary Table S1). The results consistently mirrored the expression patterns observed in both qRT-PCR and DGEs for the identified genes (Supplementary Table S1 and Figure S1). This congruence provides strong evidence supporting the robustness and reliability of the transcriptome analysis.

### 2.6. Metabolomic Analysis of 90-21-3 and D937 in Response to Exogenous NAA under K-Deficiency

**Figure 5:**
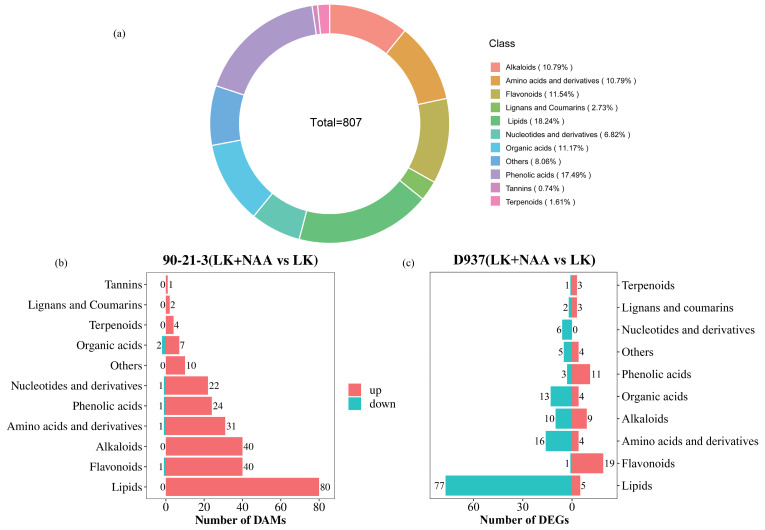
Classification of differentially accumulated metabolites (DAMs). (**a**) Classification of DAMs in general. (**b**,**c**) The classification of differential metabolites in 90-21-3 (LK + NAA vs. LK) and D937 (LK + NAA vs. LK), respectively. LK represents low potassium treatment, while LK + NAA represents low potassium stress with exogenous NAA application.

In our study of 12 samples, we identified a total of 807 differentially accumulated metabolites (DAMs) (Figure 5a). Notably, the prevalence of DAMs associated with lipids, phenolic acids, flavonoids, and organic acids was pronounced, representing 18.24%, 17.49%, 11.54%, and 11.17% of the overall DAMs, respectively. Further analysis involved the quantification of metabolites in 90-21-3 (LK + NAA vs. LK) and D937 (LK + NAA vs. LK), following stringent criteria (FC > 1.5 or FC < 0.67, and VIP > 1) (Figure 5b,c). Notably, in both comparative groups, the differential accumulation of lipids was predominant, exhibiting up-regulation specifically in 90-21-3 (LK + NAA vs. LK). Moreover, the variance in flavonoid accumulation closely followed lipids, displaying a higher upregulated pattern in both comparative groups.

### 2.7. Enrichment Analysis of Differentially Accumulated Metabolites

**Figure 6:**
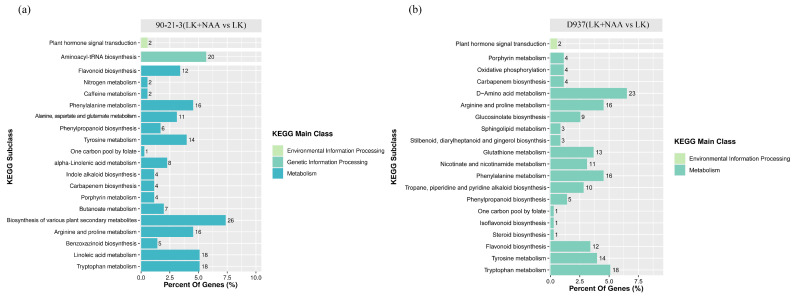
Enrichment analysis of the differential accumulation metabolites. (**a**,**b**) The top 20 KEGG pathways were enriched in the DAMs of 90-21-3 (LK + NAA vs. LK) and D937 (LK + NAA vs. LK), respectively. LK represents low potassium treatment, while LK + NAA represents low potassium stress with exogenous NAA application.

Through a comparison of DAMs in the KEGG database, we identified that the DAMs in the top 20 KEGG-enriched pathways of 90-21-3 and D937 samples were predominantly enriched within the metabolism-related pathways (Figure 6a,b). Specifically, 90-21-3 and D937 were associated with 18 and 19 metabolism-related pathways, respectively, including tryptophan metabolism, arginine, and proline metabolism, porphyrin metabolism, carbapenem biosynthesis, 1 carbon pool by folate, tyrosine metabolism, phenylpropanoid biosynthesis, and flavonoid biosynthesis, all displaying significant enrichment. Moreover, within the environmental information processing pathway classification, DAMs in both 90-21-3 and D937 samples exhibited substantial enrichment in the plant hormone signal transduction pathway. However, in the genetic information processing-related pathways, significant enrichment was observed only in 90-21-3 (LK + NAA vs. LK) within the aminoacyl-tRNA biosynthesis pathway. Based on the KEGG enrichment analysis of DEGs and DAMs, we infer that, under K-deficiency, phenylpropanoid biosynthesis plays a crucial role in the regulation of gene expression and metabolite accumulation in the roots of 90-21-3 and D937, particularly under external NAA influence. Consequently, we intend to conduct further in-depth analysis of phenylpropanoid biosynthesis, aiming for a comprehensive exploration of these pivotal pathways under K-deficiency.

### 2.8. Combined Analysis of Transcriptome and Metabolome

The identification of genes and metabolites responsive to K deficiency under LK + NAA treatment is pivotal for comprehending the inherent mechanisms governing maize root responses. Our investigation revealed significant enrichment of DEGs and DAMs in 90-21-3 and D937, particularly within the phenylpropanoid metabolism pathways.

**Figure 7:**
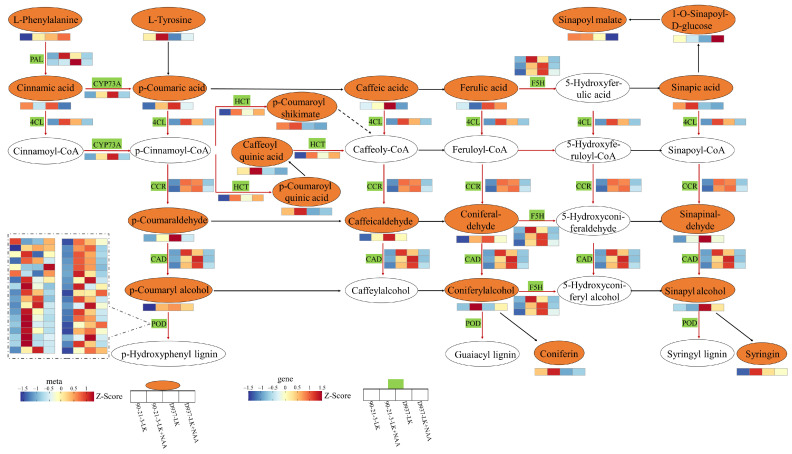
Involvement of DAMs and DEGs in the phenylpropanoid biosynthesis pathway under low K-stressed roots treated with exogenous NAA. Yellow ovals represent differential metabolites with altered content, while white ovals represent those with unchanged content. Green boxes indicate enzymes affected by differential genes. Rectangles represent differential genes or metabolites, each divided into four equal parts. The color of the rectangle reflects gene FPKM or metabolite relative abundance after *Z*-Score standardization following LK and LK + NAA treatments, as described in the scale bar. The “red solid arrow” represent genes exhibiting differential expression of regulatory enzymes, while the “black solid arrow” indicate genes of regulatory enzymes with unchanged expression levels. The “dashed arrows” represent omitted genes associated with the pathway. LK represents low potassium treatment, while LK + NAA represents low potassium stress with exogenous NAA application. PAL, phenylalanine ammonia-lyase; CYP73A, trans-cinnamate 4-monooxygenase; 4CL, 4-coumarate-CoA Ligase; HCT, shikimate O-hydroxycinnamoyl transferase; F5H, ferulate-5-hydroxylase; CCR, cinnamoyl-CoA reductase; CAD, cinnamyl-alcohol dehydrogenase; POD, peroxidase.

Based on the KEGG enrichment results, we constructed the phenylpropanoid biosynthesis pathway, which encompasses 21 DAMs and 46 DEGs (Figure 7, Supplementary Table S4). We observed that, compared to the LK treatment, LK + NAA treatment upregulated the gene expression and metabolite accumulation related to phenylpropanoid biosynthesis in 90-21-3. However, in D937, a greater number of genes were downregulated. Analysis of these DEGs revealed significant upregulation in 90-21-3 associated with the regulation of phenylalanine ammonia-lyase (PLY), trans-cinnamate 4-monooxygenase (CYP73A), 4-coumarate-CoA Ligase (4CL), shikimate O-hydroxycinnamoyl transferase (HCT), feru-late-5-hydroxylase (F5H), cinnamoyl-CoA reductase (CCR), and cinnamyl-alcohol dehydrogenase (CAD). Simultaneously, 32 out of 36 DEGs related to the regulation of peroxidase (POD) were significantly upregulated. In the metabolomic analysis, the upregulation of DEGs promoted the accumulation of related metabolites (L-Phenylalanine, Coniferyl alcohol, *p*-Coumaric acid, Caffeoylquinic acid, Syringin, *p*-Coumaryl alcohol, Coniferaldehyde, *p*-Coumaraldehyde, Sinapinaldehyde, and Caffeic aldehyde). Particularly noteworthy was the clear increase in the accumulation of L-Phenylalanine, *p*-Coumaric acid, Caffeoylquinic acid, Syringin, Caffeic aldehyde, Coniferaldehyde, *p*-Coumaryl alcohol, and Coniferyl alcohol.

In D937, DEGs involved in the regulation of PAL, CYP73A, F5H, CCR, and CAD were significantly downregulated under LK + NAA treatment. Additionally, under LK + NAA treatment, 13 metabolites (Cinnamic acid, *p*-Coumaric acid, Caffeoylquinic acid, 1-O-Sinapoyl-D-glucose, Caffeic acid, Sinapyl alcohol, *p*-Coumaryl alcohol, Coniferaldehyde, Sinapoyl malate, *p*-Coumaroyl shikimate, *p*-Coumaraldehyde, Sinapinaldehyde, and Caffeic aldehyde) in D937 exhibited significantly lower abundance compared to the LK treatment. In the phenylpropanoid biosynthesis pathway, the differential response of 90-21-3 and D937 to K-deficiency under exogenous NAA led to differential changes in the content of lignin precursors (Coniferyl alcohol, *p*-Coumaryl alcohol, and Sinapyl alcohol) in the roots.

**Figure 8:**
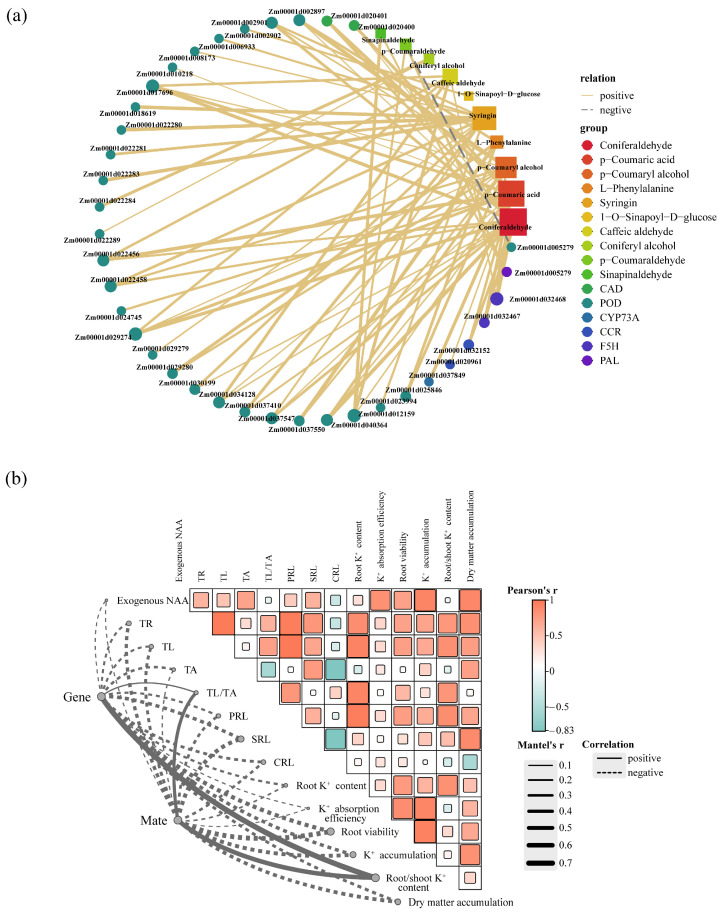
The relationship between differential genes and differential metabolites related to Phenylpropanoid biosynthesis pathway and root development. (**a**) The yellow solid lines indicate positive correlations, the gray dashed lines indicate negative correlations, the circles represent genes, and the squares represent metabolites. Node size reflects the interaction degree as indicated. (**b**) The color gradient within the network represents the Pearson correlation coefficient for pairwise comparisons. The thickness of the links connecting key genes and metabolites to root morphological and physiological indexes corresponds to the strength of the correlation coefficient (r) as determined by the Mantel test. A solid gray line signifies a positive correlation, while a dashed gray line signifies a negative correlation. TR, total root length; TL, total lateral root length; TA, total axial root length; PRL, lateral root length of primary root; SRL, lateral root length of seminal root; CRL, lateral root length of crown root; TL/TA represents the ratio of total lateral to total axial root length. “Gene” and “Mate” respectively represent the core differentially expressed genes and metabolites in the phenylpropanoid biosynthesis pathway.

To delve deeper into the interplay between DEGs and DAMs in the phenylpropanoid biosynthesis pathway, we constructed a relevant network diagram (Figure 8a). The research findings reveal a substantial correlation between 10 DAMs and 37 DEGs, predominantly displaying a positive association. Notably, genes associated with Coniferaldehyde exhibit the highest differential expression, encompassing 11 genes regulating POD, 2 genes regulating F5H, 2 genes regulating CAD, and 1 gene regulating CCR. Furthermore, secondary metabolites such as *p*-Coumaric acid (15), *p*-Coumaryl alcohol (11), and syringin (13) also exhibit a positive correlation with a substantial number of differentially expressed genes. Additionally, the principal lignin precursor in plants, Coniferyl alcohol, demonstrates a positive correlation with the genes Zm00001d022284 and Zm00001d022289, which are associated with POD. However, Zm00001d005279, involved in the regulation of POD, displays a significant negative correlation with the accumulation of *p*-Coumaraldehyde.

Based on the connectivity of DEGs and DAMs related to the phenylpropanoid biosynthesis pathway in the interaction network, we identified 11 core genes (Zm00001d002897, Zm00001d002901, Zm00001d017696, Zm00001d022456, Zm00001d022458, Zm00001d029274, Zm00001d034128, Zm00001d037547, Zm00001d040364, Zm00001d012159, and Zm00001d032468) and 7 core metabolites (Coniferaldehyde, Syringin, *p*-Coumaryl alcohol, *p*-Coumaraldehyde, Caffeic aldehyde, L-Phenylalanine, and *p*-Coumaric acid). To further elucidate the relationship between the phenylpropanoid biosynthesis pathway and root growth and development, we performed Mantel tests between core genes and metabolites with root morphological and physiological indicators. Our research results indicate a strong correlation of exogenously applied NAA with TAR, K^+^ absorption efficiency, root vitality, K^+^ accumulation, and dry matter accumulation (Figure 8b). Additionally, the root/shoot K^+^ content shows the strongest correlation with core genes and metabolites, followed by SRL, root viability, and K^+^ accumulation.

## 3. Discussion

### 3.1. Effects of Exogenous NAA on Maize Root Growth and K^+^ Uptake under K-Deficiency

Auxin plays a central role in establishing the plant root system (embryonic root formation and postembryonic root production) and in regulating root adaptation to environmental stimuli [30,31]. The developmental processes of lateral roots, which are the main organs of the plant root system for obtaining nutrients and water from the soil (localization of lateral root primordia, lateral root emergence, lateral root growth, and lateral root elongation) are also regulated by auxin [32]. Studies of K-deficiency have shown that the inhibitory effect of K^+^ on root growth is partly related to auxin signaling [33,34]. For instance, under K-deficient conditions, *Arabidopsis* can adjust root growth by modulating the degradation of PIN1, an auxin efflux carrier, and redistributing auxin within the root system [35]. However, this inhibitory effect varies between different genotypes of the same plant [36,37]. It has been found that exogenous application of auxin can alleviate the inhibitory effect of K-deficiency on root growth [38]. Tobacco research has shown that exogenous NAA application promotes the formation and elongation of lateral roots under K-deficiency [39]. In this study, we found that adding NAA promoted a significant increase in lateral roots of the K^+^-sensitive inbred line D937 under K-deficiency. Our root categorization analysis revealed that the increased lateral roots in D937 are mainly attributed to the enhancement of the embryonic root system (primary and seminal roots) [40]. In contrast, in 90-21-3, exogenous NAA primarily promoted the growth of crown roots. Although crown roots, as post-embryonic roots, play a crucial role in nutrient uptake during the later stages of maize growth, their importance during the seedling stage is relatively minor [40]. In studies on different low-K-tolerant sweet potato varieties, it was found that IAA can modulate root plasticity, promoting K^+^ uptake and dry matter accumulation in sweet potatoes under low-K^+^ stress [23]. Similarly, we also observed that exogenous NAA can enhance K^+^ absorption and dry matter accumulation in maize seedlings under K-deficiency, with a more pronounced increase in K^+^ concentration in the D937 root system. Additionally, the significant increase in root vitality upon exogenous NAA addition may be attributed to the promotive effect of exogenous NAA on root growth and development. It is noteworthy that the addition of exogenous NAA resulted in differential changes in the ratio of K^+^ between root and shoot in the two varieties. Specifically, in 90-21-3, there was an increase in K^+^ distribution in the shoot, while in D937, it promoted the migration of K^+^ toward the root.

### 3.2. Effects of Exogenous NAA on Gene Expression and Metabolite Accumulation in Maize Roots

Under K-deficiency, the absorption and transport of K^+^ in roots are closely associated with auxin transport and signaling. Previous reports have indicated the involvement of several KT/KUP/HAK family genes in auxin transport and signaling. For example, the K^+^ transporter TRH1/KUP4 participates in root hair development and geotropic responses through auxin signaling [41]. Following the application of auxin transport inhibitors, the genes responsible for K^+^ efflux transport (*KUP2*, *KUP6*, and *KUP8*) in the *Arabidopsis* root system showed an increased response to auxin, resulting in larger root cells and delayed lateral root development [42]. Here, we found that exogenous NAA promotes the transcription of genes related to proton-exporting ATPase activity, K-ion transport, and K-channel activity in the 90-21-3 root system under K-deficiency, which may enhance the K^+^ absorption efficiency and translocation to the shoot. Previous studies have shown that reactive oxygen species (ROS) act as important regulators of auxin distribution and responses in roots [43]. In the analysis of the DEGs’ GO categories, we observed a higher enrichment of antioxidant-related functions in the roots of 90-21-3. The DEGs in D937 were significantly enriched in the hormone signaling pathway, and the GO enrichment analysis was significantly enriched in the functional items related to the ethylene-activated signaling pathway (GO:0009873), cellular response to ethylene stimulus (GO:0071369), and response to ethylene (GO:0009723). Previous research has highlighted the significant role of ethylene signaling in plants under K-deficiency, including the inhibition of primary root growth, root hair elongation, ROS production, and the expression of High-Affinity K^+^ Transporter 5 (*HAK5*) [13,44]. Under K-deficiency, significant changes in Ca^2+^ levels occur in *Arabidopsis* roots, activating the CBL-CIPK signaling pathway, which further promotes K^+^ absorption or redistribution in vacuoles. In D937, we also observed the enrichment of DEGs in categories related to metal ion transport, cation transport, metal ion transmembrane transporter activity, and regulation of root development under K-deficiency after NAA addition, possibly contributing to the regulation of root elongation and K^+^ absorption.

In studies on different low K-tolerant sweet potatoes, exogenous NAA was found to enhance root responses to low-K stress by regulating auxin biosynthesis and transport, thereby increasing sweet potato tolerance to low-K stress [45]. In the analysis of DAMs, we found significant enrichment in the plant hormone signal transduction pathway and tryptophan metabolism in both 90-21-3 and D937. Flavonoids possess antioxidant properties and can stimulate the antioxidant defense system by inhibiting enzyme metabolism in the ROS generation pathway [46,47]. It has been reported that a significant increase in the levels of flavonoids, such as rutin, under salt stress can improve the effectiveness of K^+^ in quinoa leaves by scavenging hydroxyl radicals and preventing K^+^ leakage through efflux pathways [48]. In our study, the addition of exogenous NAA also led to the enrichment of DAMs in the phenylpropanoid biosynthesis and flavonoid biosynthesis pathways in maize roots under K-deficiency. Furthermore, statistical analysis of DAMs revealed the upregulation of flavonoids, which positively contributes to the enhancement of antioxidant capacity and K^+^ accumulation in maize seedling roots under K-deficiency.

### 3.3. Regulation of Exogenous NAA on Phenylpropanoid Biosynthesis Pathway under K-Deficiency

Lignin is typically composed of three precursors—Coniferyl alcohol, *p*-Coumaryl alcohol, and Sinapyl alcohol—that produce *p*-hydroxyphenyl (H), guaiacyl (G), and syringyl (S), respectively. Lignin precursors are synthesized in the cytoplasm through the general phenylpropanoid pathway into a lignin-specific pathway, which is then transported to the cell wall and polymerized into lignin [49]. Previous studies have linked reduced lignin accumulation to the expression levels of key genes in the phenylpropanoid biosynthesis pathway. *PAL*, *4CL*, *C4H*, *CCoAOMT*, *CAD*, and *CCR* are key genes for lignin synthesis in buckwheat, and their expression levels are significantly positively correlated with lignin content [50]. Among them, CCR, the first enzyme in lignin biosynthesis, converts feruloyl-CoA to conifer aldehyde, and its downregulation markedly decreases the lignin content and plant developmental rate [51]. In maize roots, *ZmCCR1* exists in adventitious roots, seminal roots, leaves, and stems, and the upregulation of the transcription level promotes lignification in these tissues [52]. F5H is involved in sinapyl alcohol formation, and mutants lacking F5H, such as the *Arabidopsis fah1* mutant, fail to produce sinapyl alcohol and its precursor sinapoyl malate [53]. CAD participates in the late steps of lignin synthesis by converting hydroxyl-cinnamaldehydes into alcohols, with minimal effects on plant lignin deposition and growth [54]. Auxin is involved in almost all aspects of plant growth and development, and lignin is also regulated by this hormone [55]. For instance, auxin response factors (ARF) 3 and ARF6 can directly bind to the promoter regions of key lignin biosynthesis genes, such as 4CL (*4CL3*, *4CL7*, and *4CL9*) or caffeoyl-CoA O-methyltransferase (CCoAOMT2), activating their expression in an auxin level-dependent manner. In studies on carrots, exogenous application of auxin (IBA) significantly improved root growth; however, at higher concentrations (100 and 150 mM IBA), it inhibited the expression of lignin-related genes, leading to reduced lignin content in roots [56]. In this study, exogenous NAA was found to regulate the transcription levels of key enzymes in the lignin biosynthesis pathway (PAL, CAD, CYP73A, CCR, F5H, and POD), showing a positive correlation with the accumulation of metabolites (L-Phenylalanine, Coniferaldehyde, *p*-Coumaric acid, *p*-Coumaryl alcohol, Syringin, 1-O-Sinapoyl-D-glucose, Caffeic aldehyde, and Sinapinaldehyde). As an important hormone for the stimulation of root development, auxin can stimulate xylem tissue differentiation [57]. Under K-deficiency, exogenous application of NAA promoted the up-regulation of most lignin synthesis-related genes and metabolites in the roots of 90-21-3 but showed a downward trend in D937. This differential response may lead to different changes in the root structure of the two varieties. Through correlation network analysis, 11 core genes were screened from the phenylpropanoid biosynthesis pathway, of which 10 genes were involved in encoding POD and were up-regulated in 90-21-3 but down-regulated in D937. Additionally, the six core metabolites screened showed the same trend. When plants are subjected to heavy metal stress, the increase in cellular lignin content is accompanied by enhanced POD activity, which helps eliminate ROS in cells, preventing oxidative damage [58,59]. Further investigation in this study revealed a significant positive correlation between the screened key genes and metabolites and the root/shoot K^+^ content. Under K-deficiency, 90-21-3 reduced the root/shoot K^+^ content under exogenous NAA treatment but exhibited the opposite trend in D937. Exogenous NAA may impact the lignin deposition in roots and the transport of water and inorganic salts by regulating the expression of POD-related genes [56,60]. However, more research is required to improve its accuracy and clarify the integration mechanism.

## 4. Materials and Methods

### 4.1. Plant Materials and Treatments

The experimental materials utilized in this study were the typical low-potassium-tolerant inbred maize line 90-21-3 and the potassium-sensitive inbred line D937, which were bred by “Reid” and “Lvdahonggu”, respectively [24,61,62]. At the seedling stage, D937 exhibited conspicuous leaf yellowing symptoms and experienced a significant reduction in plant biomass and grain yield at maturity compared to 90-21-3 under soil K-deficiency conditions. Furthermore, it adhered to all relevant institutional, national, and international standards and laws.

The experiment was conducted from July to August 2020. It took place in the solar greenhouse of the Experimental Base of Shenyang Agricultural University, Shenyang (41.8° N, 123.4° E). The experiment was performed using hydroponics. In this study, maize seeds were fully and uniformly soaked in sodium hypochlorite solution (10%, *v*/*v*) for 10 min, then rinsed four times with distilled water and finally placed in a 25 °C incubator. After 2 days, maize seeds with a germ length of 1 cm were carefully selected and subsequently grown into the two-leaf stage seedlings using the filter paper roll system [63]. The maize seedlings were grown in a sunlit greenhouse with the following climatic conditions: 16 h of light day^−1^ (200 µmol m^−2^ s^−1^), 25/16 °C (day/night). The two-leaf stage seedlings of uniform size were transferred to 3-L plastic containers filled with continuously aerated 1/2 Hoagland nutrient solution, and each container had 5 plants. This study used low-K treatment (LK, 0.2 mM KCl) as the control group and exogenous adding NAA (LK + NAA, NAA = 0.01 mM) as the experimental group. The basal nutrient solution had the following composition (μM): 2000 Ca(NO_3_)_2_, 1000 MgSO_4_, 500 NH_4_H_2_PO_4_, 100 FeNa-EDTA, 23 H_3_BO_3_, 6.3 MnSO_4_, 0.16 CuSO_4_, 0.383 ZnSO_4_, and 0.809 (NH_4_)_6_Mo_7_O_24_. All medicines were purchased from Sinopharm, Beijing, China. Each treatment was set up in triplicates. The nutrient solution was continuously aerated and renewed every 3 days. The nutrient solution’s pH was adjusted to 6.0 using 0.1 mol L^−1^ NaOH or HCl every day. We collected samples after 6 days to determine the relevant indicators (the third true leaf was fully unfolded).

### 4.2. Plant Sampling

The maize root system was categorized into crown roots (CR), seminal roots (SR), and primary roots (PR) [40], each subjected to individual scanning and analysis. Scanning was conducted using a root scanner (Epson Expression 1600 Pro, model EU-35, Tokyo, Japan) with a resolution of 600 dpi. Root images were analyzed using WinRHIZO Pro software (Version 2016c, 32 Bit, Regent Instruments Inc., Quebec, QC, Canada) to measure the total crown root length (TCR), total seminal root length (TSR), and total primary root length (TPR). The axial root lengths of CR, SR, and PR were measured using ImageJ software (version 1.45s; National Institutes of Health, Bethesda, MD, USA). The formulas for calculating the lateral root lengths of CR, SR, and PR were as follows: CRL = TCR − CRA, SRL = TSR − SRA, PRL = TPR − PRA. The total lateral root length (TL) was the sum of CRL, SRL, and PRL, while the total axial root length (TA) was the sum of CRA, SRA, and PRA. The total root length (TR) was calculated as TL + TA. The ratio of lateral root length to axial root length was determined by dividing TL by TA.

### 4.3. Potassium Content Determination

The seedlings were oven-dried at 80 °C to determine the root and shoot dry weights.

Dried samples were ground into powder and sieved through 80-mesh. A 0.3 g sample powder was then digested with 10 mL of 98% H_2_SO_4_ and 1 mL of 30% H_2_O_2_ at 300 °C for 1 h. After digestion, the solution was cooled and diluted with distilled water to a final volume of 100 mL. The K^+^ content was determined using AP1200 flame photometry (AOPU Analytical Instrument, Shanghai, China). The K^+^ accumulation, root/shoot K^+^ content, and K^+^ uptake efficiency were calculated. The K^+^ accumulation was the sum of the product of between dry weight and the concentration of K^+^ in the corresponding part of the plant. The migration coefficient of K^+^ was calculated by dividing the K^+^ content in the roots by the K^+^ content in the shoots. The K^+^ absorption efficiency was calculated by dividing the K^+^ accumulation by the dry weight of the roots.

### 4.4. Root Viability

Root vitality was determined using the triphenyl tetrazolium chloride (TTC) staining method [64].

### 4.5. Transcriptome Determination and Analysis

The roots of 90-21-3 and D937 seedlings under different treatments were collected, and three biological replicates were performed for each treatment. Liquid nitrogen was added to the samples and then transferred to a refrigerator at −80 °C for subsequent extraction of RNA. The total RNA of maize seedling roots was extracted using a RNeasy^®^ Plant Mini Kit (Qiagen, GmbH, Dusseldorf, Hilden, Germany) according to the manufacturers, and sequencing was performed by Wuhan MetWare Biotechnology (Wuhan, Hubei, China; http://www.metware.cn/, accessed on 10 December 2020). A Qubit^®^ RNA assay kit in a Qubit^®^ 2.0 fluorescence spectrometer (Life Technologies, Carlsbad, CA, USA) and RNA Nano 6000 Assay Kit on an Agilent Bioanalyzer 2100 system (Agilent Technologies, Santa Clara, CA, USA) were used to determine the quantity and quality of RNA before library preparation, respectively. Following that, next-generation sequencing libraries were constructed using the NEBNext^®^ Ultra TM RNA Library Prep Kit for Illumina^®^ (New England Biolabs, Ipswich, MA, USA) according to the manufacturer’s instructions. Finally, the cDNA library for sequencing was constructed using the Illumina Hiseq platform (Illumina, San Diego, CA, USA).

To obtain high-quality clean reads, Trimmomatic (v 0.30) was run to cut and remove containing joints, duplications, and lower sequencing quality reads. After obtaining high-quality clean reads, the clean reads were aligned with the maize reference genome using Hisat2 (v 2.0.1) software. Reference genome B73 (Zm-B73-REFERENCE-NAM-5.0) and genes annotation files were downloaded from the genome website (http://ensembl.gramene.org/Zea_mays/Info/Index, accessed on 18 December 2020). The expression level of each gene was calculated using HTSeq software (v 0.6.1) and normalized with the Reads Per Kilo bases per Million reads (RPKM) method. Additionally, the differential expression level fold-change was calculated according to the FPKM value, and the log2 (fold change) was calculated for subsequent screening of differential genes. Gene Ontology (GO, http://www.geneontology.org, accessed on 5 May 2023) and Kyoto Encyclopedia of Genes and Genomes (KEGG, https://www.genome.jp/kegg, accessed on 8 May 2023) enrichment analysis was performed for gene function and pathway analysis.

### 4.6. Metabolome Determination and Analysis

Metabolome analysis was performed by Wuhan MetWare Biotechnology (Wuhan, Hubei, China; http://www.metware.cn, accessed on 8 August 2022) using a widely targeted metabolome method. The samples used for metabolome analysis were the same batch of samples used for the transcriptome analysis. Reagents and methods for extracting metabolites were all carried out as outlined by Li et al. [65]. The ultra-performance liquid chromatography-tandem mass spectrometry (UPLC-MS/MS) method was used to obtain the present data. The detailed information on the procedure was previously described by Guo et al. [66]. The mass spectrum data were processed using Analyst Software 1.6.3 (https://sciex.com/products/software/analyst-software, accessed on 2 September 2022). Then, partial least squares discriminant analysis (PLS-DA) combined with variable importance of projection (VIP) > 1 and FC > 1.5 or FC < 0.67 was used to screen differential accumulation metabolites (DAMs). Finally, the KEGG database was used for the annotation and enrichment analysis of DAMs.

### 4.7. qPCR

The cDNA synthesis utilized the FastQuant RT Kit (TIANGEN, Beijing, China). Real-time RT-PCR was performed according to the manufacturer’s instructions of the KAPA SYBR^®^ FAST qPCR kit (KAPA Biosystems, Wilmington, MA, USA) Master Mix and QuantStudio 7 Flex (Applied Biosystems, Waltham, MA, USA). The endogenous control employed the maize *GAPDH* gene; Supplementary Table S1 comprises all the primers utilized. The 2^−ΔΔCT^ technique was adopted to assess the relative mRNA abundance.

### 4.8. Statistical Analyses

Data analyses were performed using SPSS software statistical software (Version 26; SPSS Inc., Chicago, IL, USA) and were plotted using Origin software (Version 2023; Origin Lab, Northampton, MA, USA). Different lowercase letters in the graphs indicate significant differences. Data analysis and figure preparation for the metabolome and transcriptome were performed using OmicStudio tools (https://www.omicstudio.cn/tool, accessed on 8 July 2023).

## 5. Conclusions

In this study, the root’s morphology, physiology, transcriptome, and metabolome in different low-K-tolerant maize inbred lines were comprehensively analyzed. Under K-deficiency, exogenous NAA was found to upregulate the transcription of genes related to K^+^ absorption and transport (proton-exporting ATPase, potassium transporter, cation/calcium exchanger 4, and potassium channel AKT1) as well as the synthesis of lignin and flavonoid compounds in the 90-21-3 root system. This augmentation increased the K^+^ absorption efficiency and maintained K^+^ homeostasis between root and shoot. In D937, exogenous NAA promoted the response of intercellular ethylene signaling and cation transport-related functional genes to K-deficiency, modulated the changes in hormone signaling pathway-related genes and metabolites, suppressed lignin synthesis, stimulated root elongation, and increased the root coverage area, and K^+^ content, thereby enhancing the root’s adaptation to K-deficiency conditions. This study provides novel insights into agricultural potassium management and lays the groundwork for genetic engineering and plant breeding efforts.

## Data Availability

All data in this review are publicly available.

## Supplementary Materials

The supporting information can be downloaded at: https://www.mdpi.com/article/10.3390/ijms25063366/s1.
