# Supplementary material for: Integrated Transcriptomic and Metabolomic Analysis of Exogenous NAA Effects on Maize Seedling Root Systems under Potassium Deficiency
Source: Int J Mol Sci. 2024 Mar 16;25(6):3366. doi: 10.3390/ijms25063366 (PMC10970519; doi:10.3390/ijms25063366)
Supplement: Supplementary file 1 [file ijms-25-03366-s001.zip › Table S1 & Figure S1.pdf]

Table S1. Primer list used by qRT-PCR analysis.

Gene-ID		Sequences (5'-3')
GAPDH	F	CCATCACTGCCACACAGAAAAC
	R	AGGAACACGGAAGGACATACCAG
Zm00001d002897	F	AATGGCGAGGTCCAGTGGTA
	R	GCAGGAAACGTCGTAGAAGC
Zm00001d002901	F	GGAGGATGACTTCGCCAGG
	R	GCCATGTCGGACGTGAAGAC
Zm00001d005279	F	CAGCTCAACTTGCTCAAGCG
	R	TTGTACCTCAGCTTTGGCGT
Zm00001d006933	F	TGCTTGAGCTTCAACGACCA
	R	CAGGTTGGTGTAGTAGGCGT
Zm00001d007161	F	GGCAAATGGCTTCTCAAGCG
	R	TTGCCAATCCAACCCAAGCA
Zm00001d012017	F	GCCGAATGCCTTCGACAAC
	R	ACCATGGCCTTAGCGAACTT
Zm00001d017696	F	TTCCGGAACATCCACCACAG
	R	GAAGAGCACGAGCATCTCCA
Zm00001d017762	F	TGGATCCTCCTTCCTCTCTGT
	R	CTGTCGATGACCTCGAACCC
Zm00001d024750	F	CACACTGCTCGACAATCCCT
	R	GCGAAGCACTTCTCCCTGAT
Zm00001d024751	F	CCTACGCTGAGCACCTCAAG
	R	GAGCAGTGTGTTGTCGGAGA
Zm00001d024752	F	TCCGGAACTACCTCTCGGTC
	R	GGTTGCACTTGACGATGGTG
Zm00001d028793	F	GGCCAGACTACGAAGTGGAG
	R	CCCTGACAGTGCGATCATGT
Zm00001d034128	F	GACGATCGGAGGAGCATCAG
	R	CCCGTTTCAGCTCTTTGCAC
Zm00001d036392	F	TTCGCCTCCAAGAACCTCAC
	R	GCTGAAGTTGTAGAGCCGGT
Zm00001d037547	F	TGGCAACTTCCATGGGTTGT
	R	CTGCACAATCTCCTTCGCCT
Zm00001d040364	F	CCATCACTGCCACACAGAAAAC
	R	AGGAACACGGAAGGACATACCAG

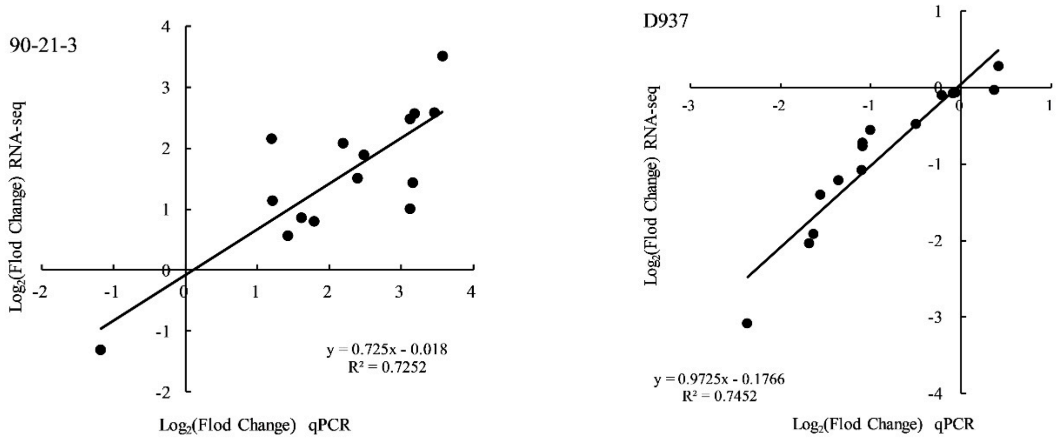

Figure S1. Correlations between qRT-PCR data and RNA-Seq profile for the 16 unigenes. Each point represents a fold change value of gene expression level in different group of 90-21-3 and D937.
